# In the name of the greater good?

**DOI:** 10.3134/ehtj.09.012

**Published:** 2010-03-31

**Authors:** K Kirkwood

**Affiliations:** School of Health Studies, Faculty of Health Sciences, University of Western Ontario, London, Ontario, Canada

## Abstract

In the event of a pandemic that poses widespread infection and high death rates, the utilitarian mandate to ‘reduce harm’ is the relevant moral value that trumps other ethical considerations. The primacy of a utilitarian approach dictates that those who are in a position to assist the cessation of the most serious outbreaks in whatever role they may have, must be present to provide their services, and those who administer health care must also be present to ensure that all responders are supported and protected to the highest degree.

Public health authorities in many economically advantaged nations are bracing themselves to face future pandemics that will harm large numbers of citizens. Modern medical horrors such as Monkeypox or the much-feared future mutations of Avian Influenza (H5N1) are mentioned in the same breath as virulent strains of influenza, as a danger to our ‘way of living.’ Far beyond sickness and large numbers of death, an outbreak of one of these pandemics poses a real threat to long-term health, as well as to the social and economic well being of significant percentages of our surviving population.^1^

While confronting issues brought forth by a pandemic, the fundamental nature of ‘public health’ and its focus on the welfare of a population demands special attention to utilitarian considerations of promotion of the greatest good—in this case, health—as well as the limitation of illness and death in the ‘worst-case’ scenarios posed by the most lethal of pandemics. Of particular interest to this paper are questions related to the obligation of health-care workers (HCWs) to report to work in the face of heightened immunological threat and whether those same workers should have greater access to immunizations and treatments than should non-HCWs.

## Utilitarianism within public health ethics

The fundamental feature of the ethical theory of utilitarianism states that moral behavior is that which promotes good and minimizes harm.^2^ In writings based on public health, utilitarianism is widely recognized as a fragment in the ethical ‘scheme’ of public health,^3^ but it is not afforded a stronger role for two primary reasons: first, considering its extreme position, utilitarianism is morally problematic,^4^ as it could literally permit anything in the name of the ‘greatest good to the greatest number,’ and second it is virtually impossible to live a moral life under the most extreme forms of utilitarianism, because the obligations are too difficult to discern (the ‘what’ of promoting the good) and impossible to execute (the ‘how’).^5^

Utilitarianism, in a moderate form, used in public health ethics, means that our actions and policies should be focused on increasing the total ‘net’ goodness rather than an average ‘net’ good for each person. The institutions of individual rights and the recognition of patient autonomy are not contradictory to this, but are believed to serve the overall good, as individual benefit increases the total good, and serves as a preventative measure of unjustified majoritarian actions against smaller groups.

This model of utilitarianism is evident in many aspects of public health—not only through health-promotion projects that encourage the otherwise illness-free individuals to take up a more healthful diet and exercise regimen but also through harm-reduction programs, in which people with negative health behaviors such as abuse of drugs or dietary fats are aided to eliminate, or at least minimize the harm they cause to those around them.

In everyday practice, the force of this utilitarian aspect has a supportive role along with other ethical elements of public health practice, and presents a balanced moral justification for all actions undertaken in accordance with this practice.^6^ However, I contend that there must be an ‘escalator clause’ in the utilitarian aspect that suggests that in the event of an extensive threat to the existence of a population, the force of this utilitarian aspect becomes the primary consideration in proportion to the threat. Therefore, the greater the threat, the greater the moral force of utilitarianism in making public health decisions. This also entails that the greater the threat, the greater the moral impetus to minimize the harm to the population.

## On duty, outbreaks, and distribution of resources

Obligations to minimize harm and promote the goods of public health are not particularly controversial in times of relatively stable ‘good-health’ measures among the populace. The more troubling question emerges from the scenario in which promoting health and minimizing illness and death demands more from HCWs—how can, or should, we compel HCWs to attend to their duties in the event that a highly lethal form of communicable disease should start spreading?^7^

Although current debates focus on questions of duty, and how much personal risk invalidates that commitment, utilitarian aspects of that obligation are not given enough weight in the debate. In many of the debates, the question of risk is posed in terms of how we do not expect a trained ‘first responder’ to recklessly endanger his or her life to save the life of another. The classic story of the lifeguard is offered as exemplar: a lifeguard is not expected to rescue a drowning swimmer if a shark is clearly present.^8^ Although this statement seems reasonable, it does not justify itself. By contrast, the consideration of the utilitarian aspect makes the point that in attempting to save a life, two are likely to be lost, thus propagating a greater total harm. The same holds true for the example of firefighters rushing into a house badly damaged by an active fire. Although there may be a life on that second floor to save, we do not expect any number of firefighters to sacrifice their lives for the doomed soul because the loss of many, including the original life in peril, is a maximization of harm, when harm should be minimized. When you control for the risks involved, such as using precautions to assure a level of safety for the rescuers, such as shark nets for the lifeguard, or safety gear for the firefighters, then the obligation to assist comes back into full force, as the potential for greater harm is manageable.^9^

It is the variable of risk, which creates variable demands on those whose duty it is to care for the population in times of crisis. We consider not only the risk to the obligated but also a question of the scope of risk to the population. In academic and public debates regarding the compulsion to attend to duty in the face of danger, one fallacy has been allowed to stand: the notion that exposure to a pandemic can be avoided if one simply does not come to his or her job as a HCW. Although it is true that working in a hospital during times of influenza outbreak puts one at a greater risk for contracting the illness,^10^ the more widespread the outbreak, the more people become sick, and the more likely the ‘stayat-home’ HCW will become sick even after having avoided contact in the course of his or her duties. We could reasonably state that, by virtue of staying home during a time of need for his or her service, the HCW improves the odds that he or she will contract this illness outside professional practice as part of the greater number who will be exposed. Another feature of the argument offered to defend dereliction of duty is to suggest that this risk that the HCW takes with his or her own health is a fixed variable, and thus should be considered as an exception to duty. On the contrary, it is a common feature of the infection-control literature that states that doctors and nurses are overwhelmingly neglectful toward their own basic infection-control protocols.^11^ Therefore, the threat is not a fixed variable, but one that is actually quite within the scope of the control of a HCW. Ethically, one cannot willfully or negligently enhance the exceptions to duty. At the same time, it is an obligation of the management to ensure that diligent HCWs are equipped to do all they can to reduce their risks. During the SARS crisis in Toronto, health-care administrators did not effectively communicate which precautions should be undertaken by HCWs to protect themselves.^12^ It bears mentioning that once clear direction could be given about the type and execution of masking procedures, the intrahospital transmission of SARS decreased to 0%.^13^ This fact speaks to the issue of risk, as the non-transmission of SARS correlated with the increased attentions of management and staff to infection-control precautions and the provision and use of proper equipment.^14^

When we speak in terms of risk and pandemics from the utilitarian perspective discussed herein, we recognize that it is completely nonsensible to sacrifice highly trained HCWs by rushing them ill equipped into dangerous situations. Much as with the example of firefighters and the unsafe burning house, we find it morally unacceptable to treat them as disposable, because of the singularity of their lives and their right to exist as individuals. There is also the detriment we would cause in an event such as a pandemic by losing the people trained to save us to the very threat they were trained to save us from. By that same logic, it could be argued that HCWs should have first access to available and medically accepted vaccinations by virtue of the fact that those HCWs are absolutely essential to our survival. The fear of an Avian Influenza outbreak brought with it much debate about scarce Tamiflu supplies and giving HCWs preferential access.^15^ However, the added value of a HCW is the fact that he or she will be facing the greater risk by virtue of faithful and responsible execution of his or her duty, and if this is true—and we have seen from the example of SARS that it is not always the case that HCWs exercise due diligence or face unmanageable risks of infection simply by being ‘on-site’—then we should do more to protect them. Nevertheless, if the claim is that they can excuse themselves from duty because of risk, then we excuse ourselves from privileging their protection, through the preferential access to measures such as Tamiflu. The same should be true for access to vaccines or treatments: those who are compelled into service to defend the overall health of a society at tremendous risk should be first in line, as their opportunity for infection—and to act as a vector for infection both within and outside their health-care facilities—means that the greater good is served by privileging their access to prophylaxis. A common objection to this comes from the perspective of social justice. The objection would point out that those who are least able to prevent their own infection, such as those from the lower socioeconomic classes, retirees and pensioners, and other vulnerable groups, would be denied access to the protections and treatments that are going to HCWs who—to varying degrees—enjoy more comfortable socioeconomic positions. Although this question of access is valid in questions of many public health interventions, the preference of HCWs in questions of preferential access to vaccines and treatments is not unjust in these terms. Fundamentally, justice addresses unjustified imbalances in treatment. Aristotle famously mandated that equals should be treated as equals, and unequals as unequals.^16^ The key point of justice is that there should be avalid justification for differential treatment, and in that light, in this context, we are describing pandemics that pose a unique and credible threat to the public in a manner that could fundamentally undermine our way of life. Preferential treatment of HCWs, in this limited context, is a just and defensible practice.

It is this same special status that we afford those who can save us from the most lethal and dangerous illnesses in times of public health emergency that also places greater demands on those same people. The greater the risk to society, the greater the responsibilities on those who can reduce the body count. The relationship between the duty of a HCW and the lethality of a disease is proportional—danger and obligation increase in step with each other, as opposed to other conceptions that suggest a threshold of exception as the risk of illness becomes too great. The fundamental flaw with this suggestion is that a negation of duty in such an outbreak simply allows the outbreak to pose an even greater threat to the population—including that same derelict HCW—rather than confronting the illness in the relatively controlled environment of a hospital.

## Conclusions

Utilitarianism in the form of promoting the good and diminishing the bad is a key moral belief in the realm of public health. It is one view in concert with others, all working to counterbalance each view to achieve a tenable moral equilibrium. In the extreme cases under consideration herein, such equilibrium dictates that the moral force of health promotion and harm minimization increases inrelation to the threat posed to the well being of a larger society. In the case of widespread death or disability caused by a pandemic, this paper contended that an increased threat generates a heightened obligation on the part of HCWs, while also creating a reasonable expectation that those same HCWs will have preferential access to vaccines and treatments.
